# Inflammasome-Independent NALP3 Contributes to High-Salt Induced Endothelial Dysfunction

**DOI:** 10.3389/fphar.2018.00968

**Published:** 2018-08-22

**Authors:** Hui Fu, Ji-Kuai Chen, Wen-Jie Lu, Yu-Jie Jiang, Yuan-Yuan Wang, Dong-Jie Li, Fu-Ming Shen

**Affiliations:** ^1^Department of Pharmacy, Shanghai Tenth People’s Hospital, Tongji University, Shanghai, China; ^2^Department of Health Toxicology, Faculty of Naval University, Second Military Medical University, Shanghai, China

**Keywords:** high salt intake, endothelial-dependent relaxation, angiogenic function, NALP3, reactive oxygen species

## Abstract

**Backgrounds and Aims:** Na^+^ is an important nutrient and its intake, mainly from salt (NaCl), is essential for normal physiological function. However, high salt intake may lead to vascular injury, independent of a rise in blood pressure (BP). Canonical NALP3 inflammasome activation is a caspase-1 medicated process, resulting in the secretion of IL-18 and IL-1β which lead to endothelial dysfunction. However, some researches uncovered a direct and inflammasome-independent role of NALP3 in renal injury. Thus, this study was designed to investigate the possible mechanisms of NALP3 in high salt induced endothelial dysfunction.

**Methods and Results:** Changes in endothelial function were measured by investigating mice (C57BL/6J, NALP3^-/-^ and wild-type, WT) fed with normal salt diet (NSD) or high salt diet (HSD) for 12W, and thoracic aortic rings from C57BL/6J mice cultured in high-salt medium. Changes of tube formation ability, intracellular reactive oxygen species (ROS), and NALP3 inflammasome expression were detected using mouse aortic endothelial cells (MAECs) cultured in high-salt medium. Consumption of HSD for 12W did not affect BP or body weight in C57BL/6J mice. Endothelium-dependent relaxation (EDR) decreased significantly in C57BL/6J mice fed with HSD for 12W, and in isolated thoracic aortic rings cultured in high-salt medium for 24 h. Results from the aortic ring assay also revealed that the angiogenic function of thoracic aortas was impaired by either consumption of HSD or exposure to high-salt medium. NALP3^-/-^ mice fed with HSD showed a relatively mild decrease in EDR function when compared with WT mice. Tube length of thoracic aortic rings from NALP3^-/-^ mice was longer than those from WT mice after receiving high-salt treatment. Inhibiting NALP3 with a NALP3 antagonist, small interfering (si) RNA experiments using si-NALP3, and decomposing ROS significantly improved tube formation ability in MAECs under high salt medium. NALP3 expression was increased in MAECs cultured with high salt treatment and inhibiting NALP3 reversed the down-regulation of p-eNOS induced by high salt in MAECs.

**Conclusion:** High salt intake impairs endothelial function, which is at least in part mediated by increasing NALP3 expression.

## Introduction

Sodium is an important nutrient that is obtained mainly from salt (NaCl). Generally, sodium consumption is essential for normal physiological function. However, high salt intake can lead to the development of essential hypertension, which is accompanied by cardiovascular and renal complications (Burnier et al., 2015). Reports have also shown that salt-sensitive hypertensive patients were susceptible to the development of myocardial hypertrophy and other cardiovascular diseases (Chamarthi et al., 2010). Studies based on surveys of sodium intake from 66 countries have shown that about 1.65 million deaths caused by cardiovascular events were due to sodium intake above the reference base of 2,000 mg/d (Mozaffarian et al., 2014).

Endothelial cells, also known as the endothelium, is a kind of single vessel layer distributing in the entire cardiovascular system. The endothelium carries out many biological functions, such as angiogenesis (forming new blood vessels), and vascular relaxation and constriction (controlling blood pressure, BP) (Flammer et al., 2012; Widmer and Lerman, 2014). The endothelium plays an indispensable role in maintaining homeostasis in a normal state. Once endothelial dysfunction occurs, a higher risk of cardiovascular diseases including hypertension follows (Liao, 2013; Matsuzawa et al., 2013). When plasma sodium concentrations increase, the endothelium is influenced by the high sodium levels and becomes more prone to stiffness, thus leading to endothelial dysfunction (Kusche-Vihrog et al., 2015). However, little is known about how high sodium leads to endothelial dysfunction.

The NALP3 inflammasome, also known as the nucleotide-binding domain and leucine-rich repeat protein-3 (NLRP3) inflammasome, is comprised of NALP3, apoptosis-associated speck-like protein (ASC), and caspase-1 (Kong et al., 2016; Deng et al., 2017; Yin et al., 2017). The NALP3 inflammasome is activated by a variety of stimuli, including ATP, uric acid crystals, low concentrations of intracellular potassium, and mitochondrial reactive oxygen species (ROS) (Martinon et al., 2006; Pétrilli et al., 2006; Zhou et al., 2011; Riteau et al., 2012). Once the NALP3 inflammasome is activated, procaspase-1 is self-cleaved to become active caspase-1 and further turn pro-interleukin-1β (pro-IL-1β) to IL-1β, which is involved in innate immune and inflammatory responses (Liu et al., 2015; Cao et al., 2017). IL-1β, a common pro-inflammatory cytokine, can lead to endothelial dysfunction (Mao et al., 2012; Mukohda et al., 2016). Chibana et al. (2017) found that endothelium-dependent vasoconstriction decreased greatly in the high IL-1β group compared with that in the low IL-1β group, and concluded that serum IL-1β levels were related to coronary endothelial dysfunction in patients with mammalian target of rapamycin (mTOR)-inhibitor-eluting stent implantation. Furthermore, anakinra, an IL-1 receptor antagonist, improves endothelial function in streptozotocin (STZ)-induced diabetic rats through reducing vascular inflammation and NADPH oxidase activity (Vallejo et al., 2014). However, Anders et al. (2017) recently confirmed a non-redundant role of NALP3 in the transforming growth factor-β (TGF-β) signaling pathway for fibroblast activation and proliferation independent of the NALP3 inflammasome complex formation *in vitro*. Similarly, Shigeoka et al. (2010) found that NALP3 played a role in renal ischemia-reperfusion injury in an inflammasome-independent manner. Wang et al. (2013) showed that a direct role of NALP3, which was independent of the inflammasome, existed in activating R-Smad and promoting TGF-β signaling in epithelial cells. These suggested that NALP3 not NALP3 inflammasome might play a role in pathophysiological conditions.

In the current work, by using mice (C57BL/6J, NALP3^-/-^, and wild-type, WT) fed with high salt diet (HSD), thoracic aortic rings from C57BL/6J, NALP3^-/-^, and WT mice by high salt treatment, and mouse aortic endothelial cells (MAECs) cultured in high salt medium, we hypothesized that NALP3 is involved in high salt induced endothelial dysfunction.

## Materials and Methods

### Animals and Treatments

Male C57BL/6J mice, aged 5–6 weeks (SLAC Laboratory Animal Ltd., Shanghai, China) were treated with normal salt diet (NSD, 0.4% NaCl) or HSD (7.0% NaCl) for 12 weeks. BP and body weight were assessed after 4, 8, and 12 weeks. Briefly, the systolic BP and diastolic BP were measured through cannulation of the right internal carotid artery under anesthesia with a combination of ketamine (100 mg/kg by intraperitoneal injection, i.p.) and midazolam (2 mg/kg, i.p.) as previously described (Liu et al., 2014). The male NALP3^-/-^ mice were a gift from Prof. Lu at the Second Military Medical University, Shanghai. After 12 weeks of NSD or HSD, NALP3^-/-^ mice and age-matched WT mice were anesthetized and sacrificed. All animals had free access to drinking water and diet. Animal maintenance and experimental procedures were in compliance with the Guide for Care and Use of Experimental Animal approved by the Animal Center, Tongji University.

### Measurement of Vascular Reactivity

Twelve weeks with NSD or HSD, mice were killed following anesthesia with sodium pentobarbital (40 mg/kg, i.p.). Thoracic aortas were harvested and the surrounding fat and conjunctive tissues were gently removed. Aortic rings were then cut into about 3 mm and placed in K-H solution. Aortic rings were required to keep a balance for 1 h when given a resting tension of 1.5 g. Finally, different concentrations of phenylephrine (Phe), acetylcholine (ACh), or sodium nitroprusside (SNP) were added, respectively, to assess endothelial function by recording changes of vascular reactivity (IOX software, EMKA Technology, Inc., Paris, France) after treatment with Phe (1 μmol/L), which was used to produce a maximal and steady contraction. ACh was used to induce endothelium-dependent relaxation (EDR), and SNP induced endothelium-independent relaxation after careful removal of the endothelium. In addition, vascular reactivity of isolated thoracic aortas from normal C57BL/6J mice were measured after 24 h incubation with normal medium (control), high salt medium (HS, 33 mmol/L NaCl), or normal medium containing 66 mmol/L mannitol that was served as an osmotic control.

### Mouse Aortic Ring Assay

The aortic ring angiogenesis assay was performed using the method reported by Baker et al. (2011) with modifications. Briefly, thoracic aortas were separated from C57BL/6J, NALP3^-/-^ and age-matched WT mice, washed with phosphate buffered saline (PBS), and cut into aortic rings (about 1.0 mm). The rings were serum-starved overnight to equilibrate their growth factor responses, effectively creating a uniform baseline state. During serum starvation, the rings were incubated in control or high salt medium for 24 h. And then the aortic rings were placed in the 24-well plates which were pre-coated with 120 μL matrigel (Corning, Berford, MA, United States), and covered with 120 μL matrigel for 10–15 min to allow matrigel polymerization. Finally, 500 μL EGM-2 medium was carefully added to the 24-well plates. Medium was changed every 2–3 days. Tube morphology was photographed using a microscope (Leica Microsystems, DMI3000B). Tube lengths of the capillary like structures were quantified using Image-Pro Plus software. We regarded the beginning incubation as day 0, and observed the angiogenesis of aortic rings every day until day 5.

### Tube Formation Assay

The MAECs (CHI Scientific, Maynard, MA, United States) were cultured in complete medium. After treatment with normal or high salt medium (NaCl, 33 mmol/L) for 6 h, cells were cultured in 96-well plates, which had been pre-incubated with 55 μL matrigel per well at 37°C for 30 min. Tube morphology was photographed with an inverted microscope. Tube lengths of the capillary like structures were quantified using Image-Pro Plus software to evaluate the angiogenic function of MAECs. The roles of glyburide (a NALP3 antagonist) and catalase (an enzyme that catalyzes H_2_O_2_ decomposition to H_2_O and O_2_) in high salt-induced tube formation capacity were evaluated.

### Intracellular Reactive Oxygen Species Measurement

Intracellular ROS levels were detected with a Reactive Oxygen Species Kit (Beyotime, Haimen, Jiangsu, China). MAECs were treated with either control or high salt medium for 6 h. Cells were then harvested and incubated with 2′,7′-dichlorodihydrofluorescein diacetate (DCFH-DA) at a final concentration of 10 μmol/L at 37°C for 20 min. Cells were washed with PBS three times, and fluorescence was detected by flow cytometry at 530 nm.

### Western Blot and ELISA

Mouse aortic endothelial cells pre-treated with different treatments were investigated. Protein concentrations were measured using a BCA Protein Assay Kit (Thermo Fisher Scientific Inc., Rockford, IL, United States) and total protein concentration was adjusted to detect the expressions of NALP3 (Cell Signaling Technology, Beverly, MA, United States), ASC (Abcam, Cambridge, United Kingdom), caspase-1 (Affinity, Cincinnati, OH, United States), cleaved caspase-1 (Cell Signaling Technology, Beverly, MA, United States), p-eNOS and e-NOS (Abcam, Cambridge, United Kingdom) by western blot. Results were analyzed using Quantity One software. The concentration of IL-1β in cell culture supernatants was determined using an ELISA kit (R&D Systems, Minneapolis, MN, United States) in accordance with the manufacturer’s instructions.

### siRNA Mediated Knock Down in MAECs

siRNA for NALP3 was obtained from GenePharma Corporation (Shanghai, China) and the siRNA transfection reagent Lipofectamine 2000 was used (Thermo Fisher Scientific Inc., Rockford, IL, United States). Briefly, MAECs were plated in 6-well plates and allowed to reach about 50% confluence. Negative control siRNA, and NALP3 siRNA in Opti-MEM medium which was mixed with Lipofectamine 2000, were added into MAECs for 6 h according to the manufacturer’s protocol. MAECs were then transferred to complete medium for another 40 h. Cells were then treated with high salt medium (33 mmol/L NaCl) for 6 h. Finally, cells were collected for western blot analysis and tube formation assay.

### Statistical Analysis

Data are shown as means ± SEM. Body weight data and results relating to the endothelial function of isolated thoracic aortas were analyzed using a two-way analysis of variance (ANOVA), followed by Bonferroni *post hoc* test. Unpaired *t*-test for two groups and one-way ANOVA for more than two groups were used for other data. A *P*-value less than 0.05 was considered statistically significant.

## Results

### High Salt Diet Had Little Impact on Blood Pressure and Body Weight in Mice

**FIGURE 1:**
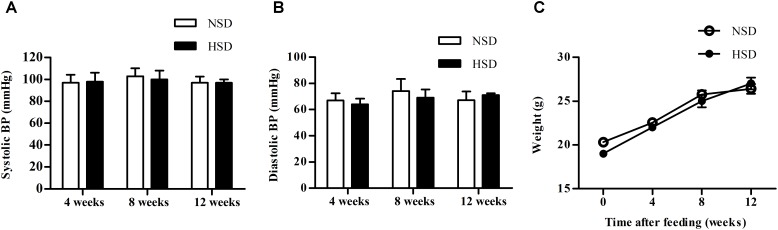
High salt diet (HSD) did not change the blood pressure and body weight in mice. C57BL/6J mice were given normal salt diet (NSD) or HSD for 12 weeks. Blood pressure (BP) and body weight were measured at 4, 8, and 12 week. **(A)** Systolic BP; **(B)** diastolic BP; **(C)** body weight. Values are shown as means ± SEM, *n* = 6 for each group in mice. **(A–C)** Two-way ANOVA followed by Bonferroni’s *post hoc* test was used and there were no interaction effects (*P* > 0.05) of diet and time on blood pressure or body weight in mice.

Blood pressure (BP) and body weight of mice fed with NSD or HSD were compared after 4, 8, and 12 weeks. No significant changes were found for both systolic and diastolic BP between the NSD and HSD groups (**Figures 1A,B**). Similarly, no significant differences in body weight were observed between two groups (**Figure 1C**).

### High Salt Treatment Decreased Endothelium-Dependent Relaxation

**FIGURE 2:**
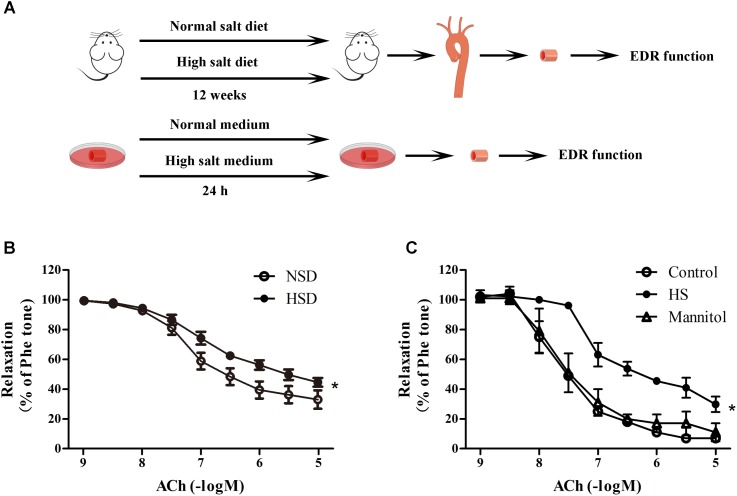
High salt treatment decreased endothelium-dependent relaxation (EDR) in thoracic aorta. **(A)** Experimental procedures in the preparation before EDR. **(B,C)** Acetylcholine (ACh) induced EDR changes in aorta treated with 12 week HSD or 24 h high salt medium (HS) incubation. Mannitol was served as an osmotic control. Values are shown as means ± SEM, *n* = 6 for each group in mice. ^∗^*P* < 0.05 vs. NSD or Control. **(B,C)** Two-way ANOVA followed by Bonferroni’s *post hoc* test was used; **(B)** there was no interaction effect (*P* > 0.05) of diet and ACh on EDR; **(C)** interaction effect (*P* < 0.01) exists in different treatments and ACh on EDR.

To investigate the effects of high salt treatment on endothelial function, EDR of thoracic aortas from mice after 12 weeks HSD or 24 h high salt incubation were measured. **Figure 2A** showed the experimental procedures used in EDR determination. It was found that 12 week HSD resulted in a significant Ach-induced EDR decrease (**Figure 2B**). In addition, it was also found that EDR was lower in aortic rings after 24 h incubation with high salt medium when compared with the control. However, aortic rings treated with mannitol, which served as the osmotic control for high salt group, showed similar EDR function as those in the control group (**Figure 2C**).

### High Salt Treatment Impaired Angiogenesis in Thoracic Aortas and MAECs

**FIGURE 3:**
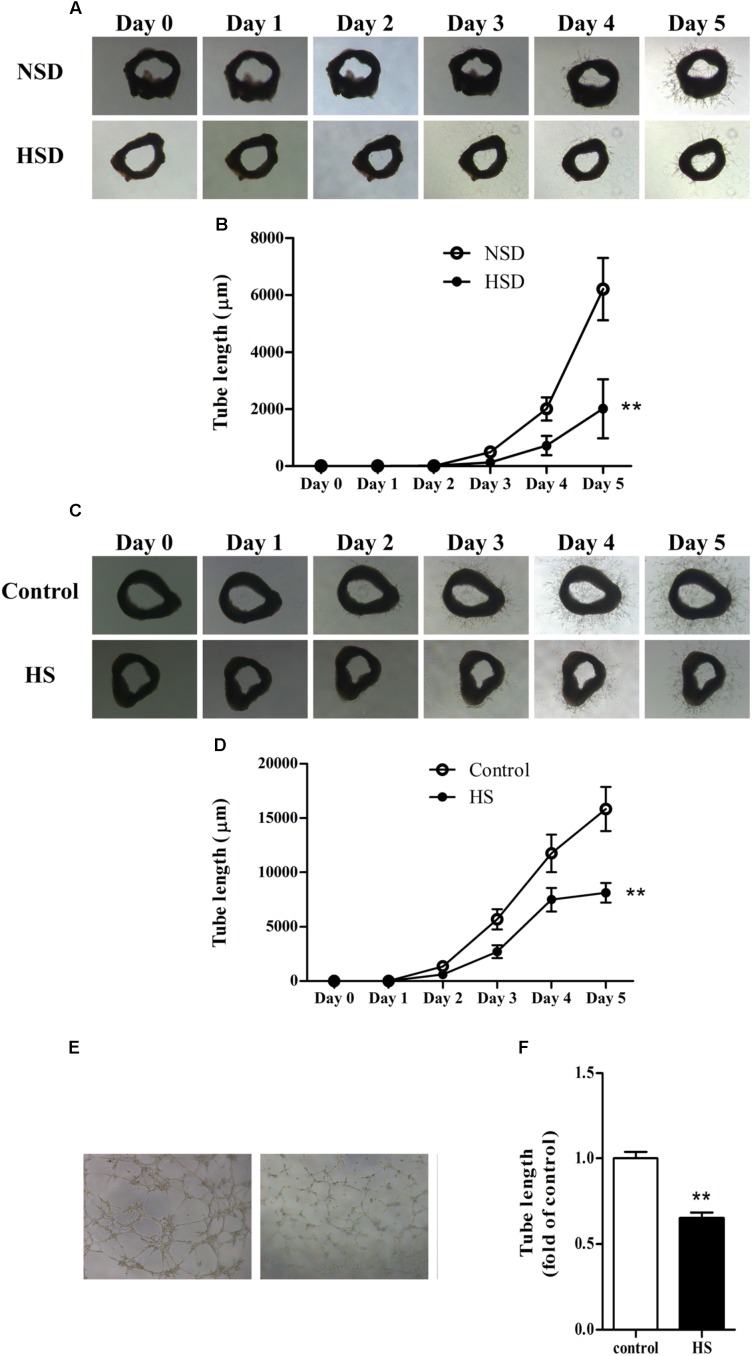
High salt impaired angiogenesis in thoracic aorta and MAECs. Representative images of microvessel sprouts **(A)** and tube length analysis **(B)** of aortic rings cultured in normal medium from C57BL/6J mice fed with normal salt diet (NSD) or HSD for consecutive 12 week, the microvessel sprouts began to merge at day 3; Representative images of microvessel sprouts **(C)** and tube length analysis **(D)** of aortic rings from C57BL/6J mice incubated with normal or high salt (HS) medium; Tube formation **(E,F)** of mouse aortic endothelial cells (MAECs) cultured in medium with 0 or 33 mmol/L NaCl (control or HS group) for 6 h. Values are shown as means ± SEM, *n* = 4 for each group in mice and *n* = 6 for each group in MAECs. ^∗∗^*P* < 0.01 vs. NSD or Control. **(B,D)** Two-way ANOVA followed by Bonferroni’s *post hoc* test was used and interaction effects (*P* < 0.01) exist in different treatments and time on the tube length of aortic rings; **(F)** Unpaired *t*-test was used.

The aortic ring assay is a well-accepted method for evaluating angiogenesis (Gimenes et al., 2017; Weng et al., 2017): thoracic aortas were isolated, cut into rings, and used in the aortic ring assay as previously described. Microvessel sprouts began to merge at day 3 in aortic rings from mice treated with either NSD or HSD for consecutive 12 week. However, vessel outgrowth was inhibited by consumption of HSD, which could be observed from the images of tube morphology. Moreover, tube lengths measured by Image-Pro Plus software revealed that total length of the microvessel sprouts was significantly decreased in aortic rings from HSD-fed mice (**Figures 3A,B**). Next, we investigated changes of angiogenesis in isolated thoracic aortas from normal mice incubated in high salt medium. Incubation in high salt medium impaired aortic angiogenesis (**Figures 3C,D**). Finally, MAECs were incubated in high salt medium for 6 h, and tube formation assay found that tube length was significantly decreased in high salt treated MAECs as compared with the control (**Figures 3E,F**).

### NALP3 Knockout and Inhibition Improved High Salt-Induced Endothelial Dysfunction

**FIGURE 4:**
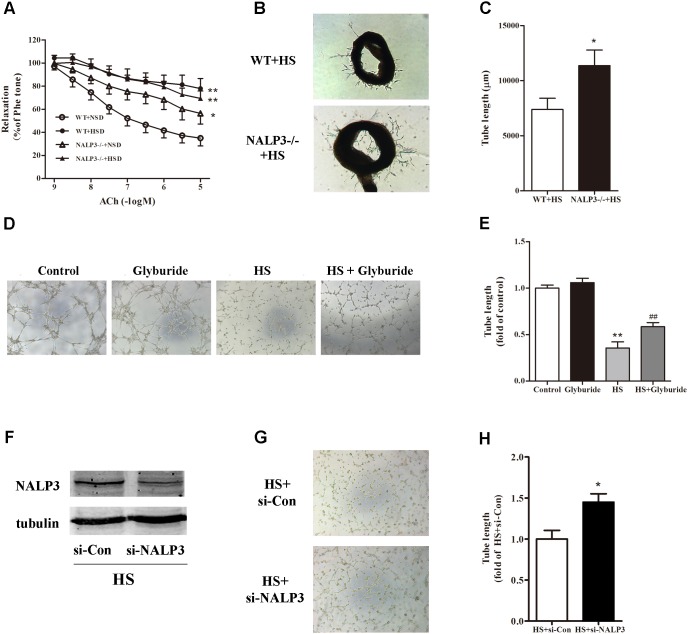
NALP3 knockout and inhibition alleviated endothelial dysfunction. NALP3^-/-^ and wild type (WT) mice were fed with normal salt diet (NSD) or HSD for 12 weeks, and then thoracic aortas were isolated to determine the endothelial function. HSD dramatically reduced acetylcholine induced EDR function in WT mice, but a slight decrease in NALP3^-/-^ mice **(A)** and total microvessel sprouts length of aortic rings in NALP3^-/-^ mice were longer than those in age-matched WT ones after high salt medium **(B,C)**; MAECs were cultured in normal, glyburide (a NALP3 antagonist), high salt (HS) or HS + glyburide medium for 6 h. Glyburide treatment significantly improved the impaired angiogenic function of MAECs by high salt **(D,E)**; Western blot analysis of whole cell lysates for the level of NALP3 expression and tube formation of MAECs were evaluated followed by small interference RNA of NALP3 with high salt medium, si-NALP3 could alleviate impaired angiogenic function of MAECs by high salt **(F–H)**. Values are shown as means ± SEM **(A)** *n* = 6 for each group in mice; **(B,C)** *n* = 4 for each group in mice; **(D,E)** *n* = 5 or 6 for each group in MAECs; **(F–H)** *n* = 4 for each group in MAECs. ^∗^*P* < 0.05 vs. NSD or WT+HS or HS+si-Con, ^∗∗^*P* < 0.01 vs. NSD or Control; ^##^*P* < 0.01 vs. HS. (**A**, Two-way ANOVA followed by Bonferroni’s *post hoc* test was used and there was no interaction effect in diet and ACh on EDR; **C,H**, Unpaired *t*-tests were used; **E**, one-way ANOVA was used).

Following activation of the NALP3 inflammasome, procaspase-1 is self-cleaved to become active caspase-1 and further turn pro-IL-1β to IL-1β, which may lead to endothelial dysfunction (Mukohda et al., 2016). Next, we investigated whether the NALP3 inflammasome played a role in high salt-induced endothelial dysfunction. Compared with WT mice, NALP3^-/-^ mice displayed a significant ACh-induced EDR decrease after fed with NSD for 12 weeks. As expected, 12 weeks HSD feeding dramatically reduced ACh-induced EDR function in WT mice when compared with WT mice fed with NSD. Interestingly, 12 weeks HSD feeding led a slight but not significant decrease of ACh-induced EDR function in NALP3^-/-^ mice when compared with NALP3^-/-^ mice fed with NSD. That is, under 10^-5^ mol/L ACh stimulation, the decrease of EDR function in NALP3^-/-^ mice was approximately 20% when comparing groups fed an HSD or NSD, whereas the decrease of EDR function in WT mice was approximately 50% (**Figure 4A**). However, NALP3 knockout altered neither Phe-induced constriction nor SNP-induced endothelial-independent relaxation (data not shown). In addition, aortic ring assay showed that the total length of microvessel sprouts at day 5 was apparently longer in aortic rings from NALP3^-/-^ mice than from WT mice cultured in high salt medium (**Figures 4B,C**). To further confirm the role of NALP3 in HS induced endothelial dysfunction, we conducted a NALP3 antagonist and small interference RNA of NALP3 *in vitro*. It was found that high salt impaired the tube formation ability of MAECs, and glyburide (a NALP3 antagonist) could significantly improve the impaired angiogenic function of MAECs (**Figures 4D,E**). Western blot analysis showed that NALP3 expression was down-regulated under high salt medium conditions when pre-treated with si-NALP3 (**Figure 4F**). Meanwhile, the tube formation ability was improved in MAECs treated with si-NALP3 after high salt treatment (**Figures 4G,H**). These results indicated that NALP3 plays a role in high salt-induced endothelial dysfunction.

### Increased ROS Production by High Salt Contributed to Endothelial Dysfunction

**FIGURE 5:**
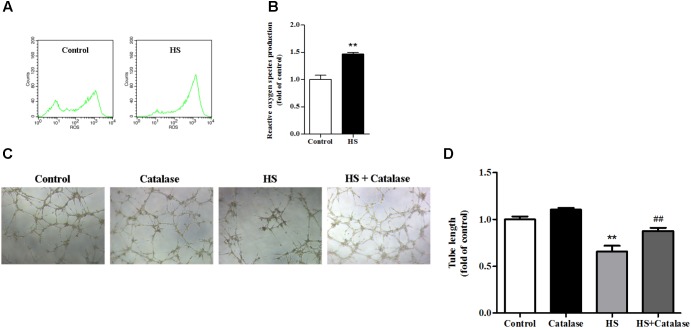
Increased ROS production by high salt contributed to endothelial dysfunction. MAECs were cultured in normal, or high salt (HS), or HS + Catalase (a H_2_O_2_ catalyzing enzyme) medium. **(A,B)** HS induced a higher level of ROS production in MAECs, *n* = 4 for each group. **(C,D)** Catalase significantly improved the impaired angiogenic function by HS, *n* = 5 for each group. Values are shown as means ± SEM. ^∗∗^*P* < 0.01 vs. Control, ^##^*P* < 0.01 vs. HS. (**B**, Unpaired *t*-test was used; **D**, one-way ANOVA was used).

A previously study has proved that the NALP3 inflammasome may work as a sensor for metabolic danger and may be activated by ROS (Schroder et al., 2010). In this study, we found that, compared with the control, intracellular ROS production in MAECs was significantly increased (**Figures 5A,B**). It is well known that the major component of ROS is H_2_O_2_, which can be decomposed to H_2_O and O_2_ by catalase. After catalase treatment, the reduced tube formation ability of MAECs caused by high salt was significantly improved (**Figures 5C,D**). These results suggested that the increase of ROS caused by high salt contributed to endothelial dysfunction.

### High Salt Up-regulated NALP3 Expression and Inhibiting NALP3 Reversed the Down-Regulated Expression of p-eNOS Induced by High Salt in MAECs

**FIGURE 6:**
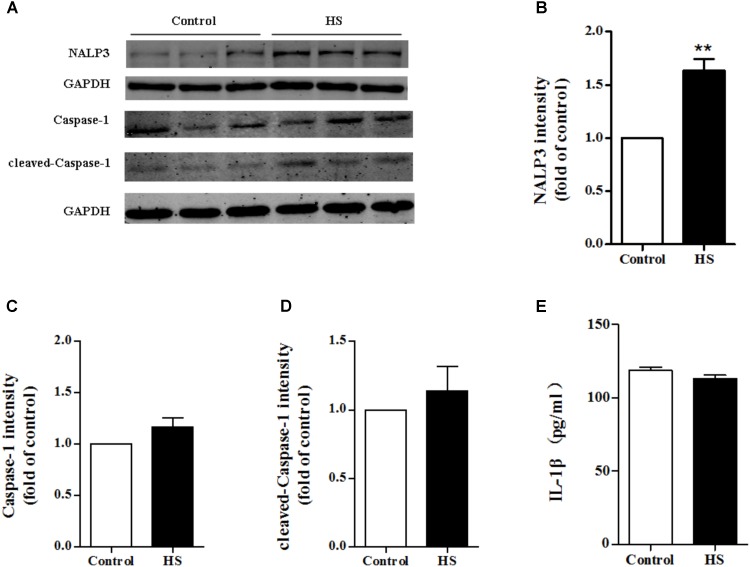
High salt up-regulated NALP3 expression. MAECs were treated in normal or high salt (HS) medium for 6 h, and then cell or cell supernatant were collected to detect the expression of NALP3 **(A,B)**, Caspase-1 **(C)**, cleaved caspase-1 **(D)** and IL-1β **(E)**. Values are shown as means ± SEM **(A–D)** *n* = 3 for each group; **(E)** *n* = 6 for each group ^∗∗^*P* < 0.01 vs. Control. (**B–E** Unpaired *t*-tests were used).

As high salt increased ROS production, we next investigated whether high salt could lead to NALP3 inflammasome activation, and subsequently change the expression of caspase-1 and IL-1β. Compared with the control, the expression of NALP3 increased significantly in MAECs cultured in high salt medium (**Figures 6A,B**). However, little changes of caspase-1, cleaved caspase-1 and IL-1β expression were found (**Figures 6C–E**). It was also found that the protein levels of ASC were not changed after HS treatment (**Supplementary Figure 1**). Furthermore, we measured the protein levels of NALP3 with catalase treatment to validate the regulated role by ROS. Reducing ROS production with catalase partly blocked the up-regulation of NALP3 induced by high salt in MAECs (**Supplementary Figure 2**).

**FIGURE 7:**
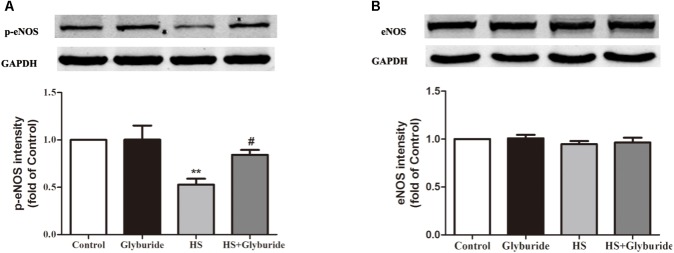
Glyburide reversed the effect of high salt on the expression of p-eNOS in MAECs. MAECs were cultured in normal, glyburide, high salt (HS) or high salt + glyburide medium (HS + glyburide) for 6 h. **(A)** HS down-regulated p-eNOS expression in MAECs and the effect could be reversed by HS + glyburide. *n* = 6 in each group. **(B)** Glyburide had little effect on the expression of eNOS in MAECs. *n* = 4 in each group. Values are shown as means ± SEM. ^∗∗^*P* < 0.01 vs. Control, ^#^*P* < 0.05 vs. HS. (**A,B** one-way ANOVA were used).

Due to the increased expression of NALP3 with high salt treatment, we used glyburide (a NALP3 antagonist) to block the role of NALP3 in MAECs. It was found that high salt could down-regulate p-eNOS expression and this effect could be reversed by high salt + glyburide (**Figure 7A**), while Glyburide itself had little effect on the expression of eNOS in MAECs with or without high salt treatment (**Figure 7B**).

## Discussion

The main findings of the present study are: (1) High salt treatment led to endothelial dysfunction in C57BL/6 mice, in isolated thoracic aortic rings from C57BL/6 mice, and in MAECs; (2) high salt-induced endothelial dysfunction was alleviated in NALP3^-/-^ mice and in MAECs by either down-regulating NALP3 or decomposing H_2_O_2_; (3) high salt increased NALP3 expression in MAECs and inhibiting NALP3 reversed the down-regulated expression of p-eNOS induced by high salt in MAECs.

Consumption of an HSD is associated with hypertension and increased rates of other cardiovascular diseases (Nichols et al., 2015; Lankhorst et al., 2017; Pavlov et al., 2017). Sodium, as one of the essential nutrients in the body, is mainly obtained from salt intake. The physiological need for sodium is approximately 500 mg/day, and an adequate intake of sodium is set at a conservative value of 1,500 mg/day (Frieden, 2016). Reducing sodium consumption could gradually decrease risks of stoke, myocardial infarction, and other complications (Frieden, 2016). Though high BP is closely linked with HSD, increasing evidences clearly indicated that high dietary salt intake represents a risk factor (independent of increased BP) for the development of cardiovascular disease (de Wardener et al., 2004; Liu et al., 2014). In this study, we found that consumption of an HSD for 4, 8, and 12 weeks did not lead to increased BP in mice.

Endothelial cells act as a barrier to separate blood from underlying tissue and are involved in many important processes of vascular biology, including inflammation, angiogenesis (formation of new blood vessels), as well as vasoconstriction and vasodilation (BP control) (Flammer et al., 2012). Endothelial dysfunction, thought to be a primary causative event in the development of atherosclerosis, predicts cardiovascular outcomes independent of conventional cardiovascular risk factors (Veerasamy et al., 2015). Studies have demonstrated that an HSD (6,900–8,050 mg sodium/day for 7 days) impaired endothelium-dependent dilation, which was independent of changes in BP (DuPont et al., 2013), and that long term high salt intake caused a significant reduction in aortic relaxation in response to ACh (Barton et al., 1998). In this work, we found that both consumption of an HSD (7% NaCl) in mice and exposure of isolated thoracic aortic rings to high salt medium (33 mmol/L NaCl) impaired endothelial function as reflected by either EDR measurement or an aortic angiogenesis assay, and that incubating MAECs with high salt medium (33 mmol/L NaCl) impaired endothelial function as reflected by tube formation assay. These results suggested that high salt treatment could lead to endothelial dysfunction.

The NALP3 inflammasome, a sensor for metabolic danger (Schroder et al., 2010), takes part in innate immune response and is critical for the regulation of the inflammatory cytokine IL-1β, which can lead to endothelial dysfunction (Mukohda et al., 2016). Loughrey et al. (2003) found that EDR of the aorta from Sprague-Dawley rats rapidly decreased after stimulation with IL-1β for 2 h, while an IL-1 receptor antagonist could improve impaired endothelial function in streptozotocin-induced diabetic rats (Vallejo et al., 2014). In this work, we investigated whether the inflammasome component NALP3 or NALP3 inflammasome would be involved in high salt-induced endothelial dysfunction. The sulfonylurea drug glyburide, usually used clinically to treat type II diabetes, is a non-selective antagonist of NALP3 and could prevent activation of the NALP3 inflammasome and crystal-induced IL-1β secretion (Lamkanfi et al., 2009). Glyburide also improved impaired tube formation ability of MAECs caused by high salt stimulation. Importantly, by using NALP3-knockout mice, we confirmed that the reduction of EDR by HSD could be relieved, as compared with that in WT mice. That is, under 10^-5^ mol/L ACh stimulation, the decrease of EDR function in NALP3^-/-^ mice was approximately 20% when comparing groups fed with HSD or NSD, whereas the decrease of EDR function in WT mice was approximately 50%. Furthermore, NALP3 knockout or interference could improve angiogenic function reduced by high-salt treatment. These results indicated the involvement of inflammasome component NALP3 in high salt-induced endothelial dysfunction.

Reactive oxygen species takes part in many damage and stress sensing processes and its production is a highly conserved signal. Previous studies have suggested that ROS may activate the NALP3 inflammasome, and that ROS scavengers can block this process, suggesting that ROS is an upstream regulator of NALP3 inflammasome activation (Schroder et al., 2010; Lee, 2011; Ma et al., 2014). The major component of ROS is H_2_O_2_, which can be decomposed to H_2_O and O_2_ by catalase. In this study, we found that, compared with the control, intracellular ROS production in MAECs after high salt treatment was significantly increased, while tube formation ability was decreased. Catalase treatment improved impaired endothelial function induced by high salt. In addition, high salt up-regulated NALP3 expression in MAECs, and the effect was partly abolished by catalase + high salt. It is in agreement with the previous study (Hoyt et al., 2017) that ROS production triggers NALP3 activation. Together with the results that NALP3 knockout and inhibition alleviated endothelial dysfunction, we proposed that increased ROS production by high salt contributed to activation of the NALP3, and led to endothelial dysfunction.

Activation of the NALP3 inflammasome leads to cleavage of pro-caspase-1 into caspase-1, and activated caspase-1 then cleaves pro-IL-1β to IL-1β to participate in inflammatory processes (Zielinski et al., 2017). In this work, we found that high salt treatment up-regulated NALP3 expression in MAECs. We also determined the expression of ASC, pro-caspase-1, cleaved caspase-1, and IL-1β. However, the downstream products of NALP3 were not altered when comparing the control and high salt-treated MAECs. Xiao et al. (2017) reported that, isoproterenol induced inflammasome-dependent activation of IL-18 in myocardium, but not IL-1β. Thus we measured the expression of IL-18 in the supernatant of MAECs. However, IL-18 were not detected in the supernatant of MAECs either in control or high salt medium in our study. Recently, Anders et al. (2017) reported a non-redundant role of NALP3 in the TGF-β signaling pathway for fibroblast activation and proliferation independent of the NALP3 inflammasome complex formation. Although it is well accepted that the ROS/NALP3 inflammasome/IL-1β signaling pathway is able to induce endothelial dysfunction (Loughrey et al., 2003; Schroder et al., 2010; Mukohda et al., 2016), we postulate that there may be other pathways for NALP3 to induce endothelial dysfunction, independently of the NALP3 inflammasome complex formation. Previous studies (Thum et al., 2007; Yu et al., 2016) have suggested that the dysfunction of endothelial progenitor cells in diabetes was the loss of protection of NO due to reduced synthesis from eNOS. Thus we measured the protein levels of p-eNOS/eNOS to explore whether Glyburide (a NALP3 antagonist) improved endothelial function by inhibiting NALP3. It was showed that high salt reduced p-eNOS expression in MAECs and inhibiting NALP3 with glyburide reversed the down-regulation of p-eNOS with high salt treatment.

## Conclusion

High salt could lead to increased NALP3 expression and endothelial dysfunction, and inhibiting NALP3 alleviates endothelial dysfunction.

## Author Contributions

HF and J-KC performed the experiments and analyzed the data. HF drafted the manuscript. W-JL and Y-JJ assisted and Y-YW supported in the experiments. D-JL participated in the data analysis and the design of this study. F-MS revised the paper and gave important advice to the study. All authors contributed to the writing of the manuscript, read, and approved the final editing.

## Conflict of Interest Statement

The authors declare that the research was conducted in the absence of any commercial or financial relationships that could be construed as a potential conflict of interest.
