# Supplementary material for: Inflammasome-Independent NALP3 Contributes to High-Salt Induced Endothelial Dysfunction
Source: Front Pharmacol. 2018 Aug 22;9:968. doi: 10.3389/fphar.2018.00968 (PMC6113916; doi:10.3389/fphar.2018.00968)
Supplement: Supplementary file 1 [file Table_1.docx]

**Inflammasome-independent NALP3 contributes to high-salt induced endothelial dysfunction**

**Running title**: NALP3 contributes to endothelial dysfunction

*Hui Fu1, Ji-Kuai Chen2, Wen-Jie Lu1, Yu-Jie Jiang1, Yuan-Yuan Wang1, Dong-Jie Li1*, Fu-Ming Shen1**

*1Department of Pharmacy, Shanghai Tenth People's Hospital, Tongji University, Shanghai, China*

*2Department of Health Toxicology, Faculty of Naval Medicine, Second Military Medical University, Shanghai, China.*

*Correspondence to these authors:

**Fu-Ming Shen**

Department of Pharmacy

Shanghai Tenth People's Hospital, Tongji University

301 Middle Yanchang Road, Shanghai 200072, China

Tel: +86-021-66307668, E-mail: [fumingshen@tongji.edu.cn](mailto:fumingshen@tongji.edu.cn)

**Dong-Jie Li**

Department of Pharmacy

Shanghai Tenth People's Hospital, Tongji University

301 Middle Yanchang Road, Shanghai 200072, China

Tel: +86-021-66302570, E-mail: [djli@tongji.edu.cn](http://mailto:djli@tongji.edu.cn/)

**Supplementary Figures:**

**
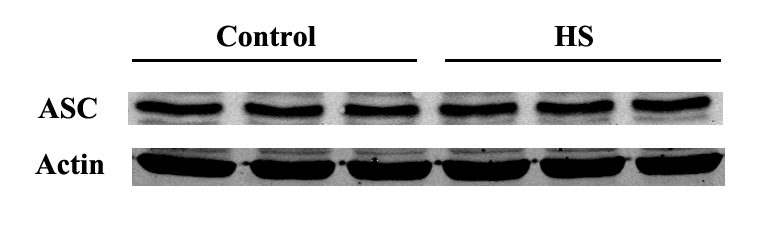
**

**Supplementary Figure1. High salt did not alter the ASC expression in MAECs.** MAECs were treated in normal and high salt medium (NaCl, 33 mmol/L) for 6h, and then were collected to detect the expression of ASC. n=6 in each group.


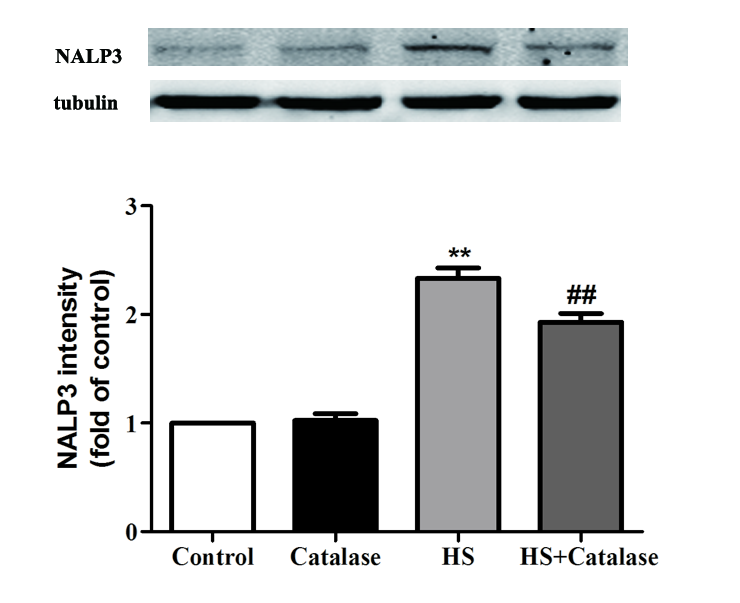


**Supplementary Figure2. Catalase blocked the up-regulation of NALP3 induced by high salt in MAECs.** MAECs were cultured in normal, high salt (HS), or HS + Catalase (a H2O2 catalying enzyme) medium for 6h and then cells were collected to determine the expression of NALP3. Values are shown as means ± SEM. ** P < 0.01 vs Control, ## P < 0.01 vs HS. (one-way ANOVA was used)
